# *CDKN2A/B* status versus morphology in diagnosing WHO grade 4 *IDH*-mutated astrocytomas: what is the clinical relevance?

**DOI:** 10.1007/s11060-025-05078-x

**Published:** 2025-05-20

**Authors:** Anna Lipatnikova, Teresia Kling, Anna Dénes, Louise Carstam, Alba Corell, Malin Blomstrand, Sandra Ferreyra Vega, Dima Harba, Thomas Olsson Bontell, Helena Carén, Asgeir S. Jakola

**Affiliations:** 1 Department of Clinical Neuroscience, Institute of Neuroscience and Physiology, Sahlgrenska Academy, University of Gothenburg, Blå stråket 7, Floor 3, Gothenburg, 413 45 Sweden; 2 Sahlgrenska Center for Cancer Research, Department of Medical Biochemistry and Cell Biology, Institute of Biomedicine, Sahlgrenska Academy, University of Gothenburg, Gothenburg, Sweden; 3 Department of Neurosurgery, Sahlgrenska University Hospital, Gothenburg, Sweden; 4 Department of Physiology, Institute of Neuroscience and Physiology, Sahlgrenska Academy, University of Gothenburg, Gothenburg, Sweden; 5 Department of Oncology, Institute of Clinical Sciences, Sahlgrenska Academy, University of Gothenburg, Gothenburg, Sweden; 6 Department of Clinical Pathology, Sahlgrenska University Hospital, Gothenburg, Sweden

**Keywords:** CDKN2A/B, IDH-mutant astrocytoma, WHO grade 4, DNA methylation

## Abstract

**Purpose:**

In the 2021 WHO classification system for central nervous system tumors, the diffuse glioma subgroup *IDH*-mutated (IDHm) astrocytomas WHO grade 4 was introduced. The diagnosis can be based upon molecular or histopathological morphological criteria. Here we explore whether phenotype and survival of IDHm astrocytomas WHO grade 4 differed across the criteria used for diagnosis.

**Methods:**

Patients with IDHm astrocytoma, WHO grade 4, were included from Sahlgrenska University Hospital and TCGA database. We created three subgroups based upon the criteria for diagnosis of WHO grade 4; (1) homozygous *CDKN2A/B* deletion; (2) morphological (necrosis and/or microvascular proliferation); (3) combined subgroup with both homozygous *CDKN2A/B* deletion and morphological grade 4 criteria.

**Results:**

We included 90 patients (local cohort, *n* = 35, TCGA cohort, *n* = 55) with IDHm astrocytoma, WHO grade 4. The median survival was 4.1 years (95% CI 3.0-5.3). Survival was comparable when the diagnosis was based on homozygous *CDKN2A/B* deletion and on morphological WHO grade 4 criteria (5.2 vs. 5.3 years). However, in the combined subgroup, survival was significantly shorter (2.8 years, *p* = 0.006).

**Conclusion:**

The different subgroups of IDHm astrocytoma WHO grade 4 share similar characteristics. Patients whose tumors exhibit combined criteria have worse prognosis.

**Supplementary Information:**

The online version contains supplementary material available at 10.1007/s11060-025-05078-x.

## Key points

- IDHm grade 4 astrocytomas with both *CDKN2A/B* homozygous loss and microvascular proliferation and/or necrosis have worse prognosis.
- Patients with morphological criteria fulfilled and those with *CDKN2A/B* homozygous loss have comparable overall survival.

**Supplementary Information:**

The online version contains supplementary material available at 10.1007/s11060-025-05078-x.

## Importance of study

*IDH*-mutated (IDHm) astrocytoma WHO grade 4 is a newly introduced and rare subgroup of adult-type diffuse astrocytomas with limited research exploring its overall survival (OS) and baseline characteristics. Moreover, few studies have investigated how genetic and morphological features within IDHm astrocytoma WHO grade 4 influence OS and clinical characteristics. This study explored if the different diagnostic criteria for IDHm astrocytoma WHO grade 4 have implications in terms of survival and if this tumor type could arise through distinct biological pathways. Refined insights to prognosis could inform clinical decision-making, inform clinical trial designs and improve patient outcomes.

**Supplementary Information:**

The online version contains supplementary material available at 10.1007/s11060-025-05078-x.

## Introduction

In the 2021 World Health Organization (WHO) classification system, the diagnosis of IDHm astrocytoma WHO grade 4 was introduced. This diagnosis can be established based either on molecular or morphological criteria. The molecular WHO grade 4 criteria are defined as the presence of a *CDKN2A/B* homozygous deletion (*CDKN2A/B* deletion) while the morphological criteria are based on the presence of microvascular proliferation and/or necrosis [1].

This classification suggests that, in theory, there could be three subgroups of IDHm astrocytoma WHO grade 4; those exhibiting only molecular WHO grade 4 criteria (*CDKN2A/B* deletion), those with only morphological WHO grade 4 criteria and those presenting with combined criteria.

The literature is sparse concerning the importance of the different diagnostic means and whether the different criteria are associated with clinical differences in demographics, baseline characteristics or survival. Direct comparison is limited but the median overall survival in morphological WHO grade 4 IDHm astrocytomas is reported to range between 0.9 and 5.3 years [2–6]. In patients with tumors harboring *CDKN2A/B* deletion, but lacking the morphological grade 4 characteristics, the median survival is in the range 3.1-5.0 years [2, 7, 8].

Concerning the combined criteria, *CDKN2A/B* deletion is indeed more frequent in IDHm astrocytomas with morphological WHO grade 4 criteria compared to the morphological lower-grade astrocytomas [4, 9]. The prognostic negative impact of *CDKN2A/B* deletion in IDHm astrocytomas with morphological lower-grade features is established and the reason for the molecular criteria of WHO grade 4 astrocytomas [2, 8, 10–12]. However, there is inconsistent data concerning the prognostic impact of *CDKN2A/B* deletion in patients already presenting with morphological characteristics of WHO grade 4 astrocytoma [2, 4, 10–12].

Due to the conflicting findings, the present study was conducted to investigate survival in patients with IDHm astrocytoma WHO grade 4 based upon the different diagnostic criteria. In addition, we compared patient, tumor and treatment characteristics within these subgroups.

## Materials and methods

### Study design

This retrospective study defined the following criteria for inclusion: (i) having undergone primary tumor resection in the period 2007–2022 at Sahlgrenska University Hospital in Gothenburg, Sweden, (ii) diagnosis of IDHm astrocytoma WHO grade 4 as defined by the WHO 2021 classification system and (iii) age above 18 years at initial diagnosis. We created three subgroups by the different criteria for diagnosis of IDHm astrocytoma WHO grade 4: (1) presence of *CDKN2A/B* deletion (molecular diagnosis), (2) presentation with necrosis and/or microvascular proliferation (morphological diagnosis) and (3) a combined subgroup where both molecular and morphological criteria were fulfilled.

### Public dataset

Patients with IDHm gliomas from The Cancer Genome Atlas (TCGA) (TCGA-GBM and TCGA-LGG) were identified. These patients were further screened for *CDKN2A/B* deletion and/or morphological WHO grade 4 criteria as outlined below. The public dataset was only used for survival analysis.

Morphological grading was assessed for all patients by reviewing pathology reports in cBioPortal to ensure accurate classification [13].

### Study population and data collection

A database containing patients with diffuse gliomas operated at Sahlgrenska University Hospital between 2007 and 2022 was screened to identify IDHm astrocytomas WHO grade 4. Clinical and pathological data were collected from medical records. End of follow-up was set to November 1st 2023. Tumor volume pre- and postop was measured using 3D slicer version 5.6.2 [14]. When ring-like enhancement was present (and not obviously constituting a smaller part of a larger non-enhancing tumor), T1Gd was used for segmentation. Otherwise T2w images (T2 or FLAIR) were used. All scans included in the subgroup analyses were reviewed by an experienced neurosurgeon to determine the appropriate image modality for segmentations. Examples of segmentations are provided in supplementary material Fig. 1.

### Molecular assessment

IDHm status was determined with methods described in detail previously [15], but in short, included DNA extraction from formalin-fixed paraffin-embedded (FFPE) tumor tissue samples, except in one case where fresh-frozen tissue was used due to unavailability of a FFPE sample. Cases with unavailable tissue were excluded.

Genome-wide DNA methylation profiles were generated using Illumina Infinium MethylationEPIC arrays (v1 and v2). Cases with poor DNA quality insufficient for evaluation were excluded, except when histological examination revealed microvascular proliferation and/or necrosis. In such instances, the patient was included in the analysis of IDHm astrocytomas WHO grade 4 as a whole group but excluded from comparisons of survival and characteristics across different subgroups.

The G-CIMP phenotype was determined according to Ceccarelli et al. [16] using the TCGA biolinks R package [17]. *IDH* mutations were confirmed using Sanger sequencing [15]. O-6-Methylguanine-DNA Methyltransferase (MGMT) status was retrieved from clinical records, and in cases of missing data, methylation-based assessment was performed [15]. To evaluate *CDKN2A/B* status, DNA-methylation profiles from the local cohort were generated by methods described in earlier publications [15, 18]. Copy number alterations (CNA) profiles for each individual tumor sample were established from raw methylation data using the R software with R studio (version 4.0.2) and the package conumee [19]. A *CDKN2A/B* homozygous deletion was determined by the cutoff <-0.4 [11, 20].

Bulk tumor DNA methylation array data (Infinium HumanMethylation450 BeadChip or EPIC) from TCGA were downloaded and screened for *CDKN2A/B* deletion with methods described above. In cases where methylation data were unavailable in TCGA, *CDKN2A/B* status was retrieved from cBioPortal (PanCancer Atlas), where status was assessed using genomic copy number data analysed by the GISTIC2.0 algorithm [21]. A GISTIC value of ≤-2 denoted a homozygous deletion [22].

### Statistics

The descriptive statistics provided in this paper was calculated using R (version 4.4.1). The flow chart provided in the paper was made using PowerPoint (version 16.95.4). Crosstabulations were created to summarize the relationship between categorical variables, and Fisher’s Exact test was applied to assess statistical significance for dichotomous variables. When analyses were made between 3 groups with continuous data, the Kruskal-Wallis test was applied since data was skewed.

Overall survival was estimated by the Kaplan-Meier method (curves plotted with R-package “survminer” [23]). Log-rank tests were run to compare survival curves, using the “survival” package in R [24]. P-values less than 0.05 were considered significant.

## Results

**Fig. 1:**
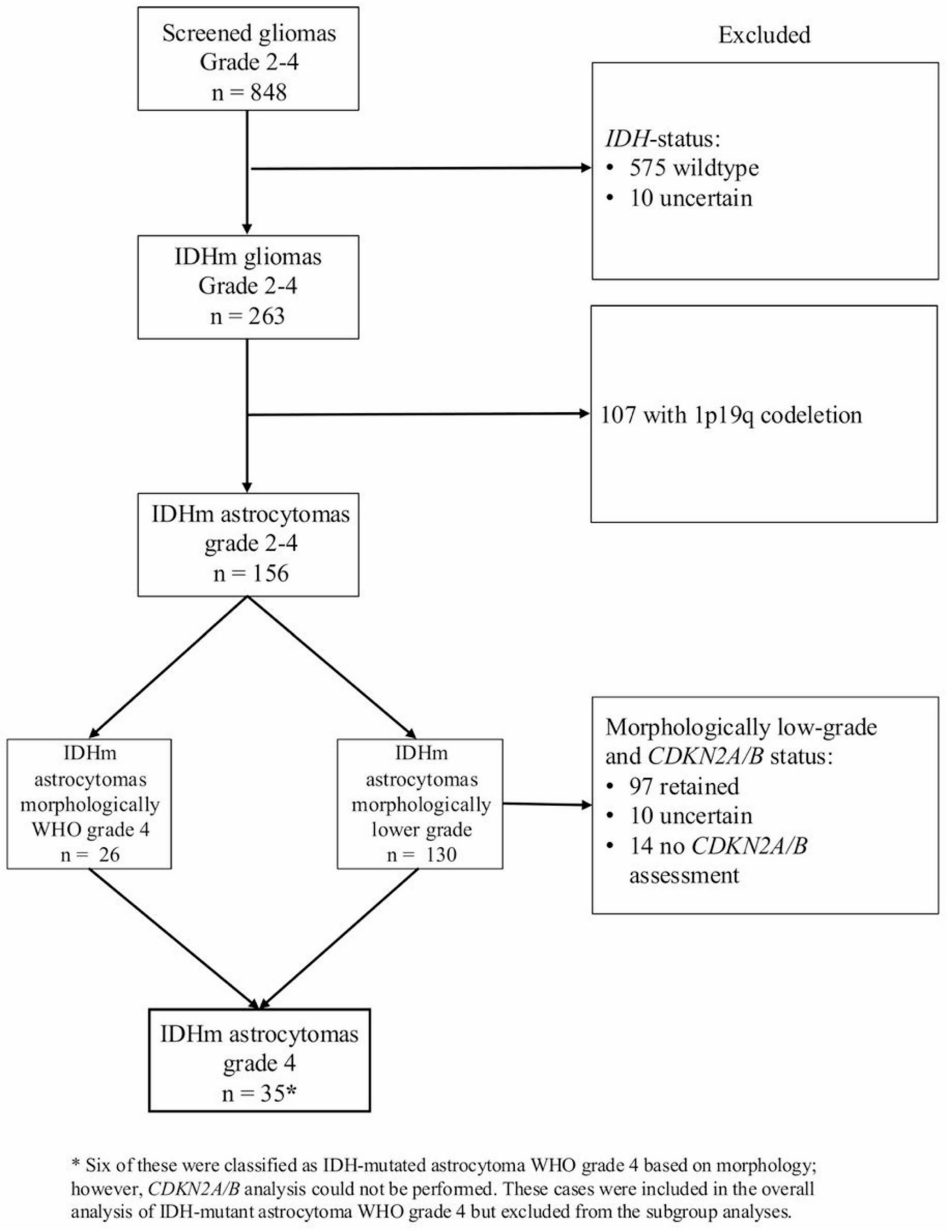
Flowchart of patients screened for this study. Patients excluded from *CDKN2A/B* analysis were either removed due to poor tissue quality or unavailable tissue at time of assessment. Tumors with morphological WHO grade 4 criteria, but no *CDKN2A/B* assessment, were included based on their morphological features but excluded from the characteristic’s comparison between subgroups

From a total of 848 adult patients who underwent surgery for diffuse gliomas at Sahlgrenska University Hospital between 2007 and 2022 we included 35 patients with WHO grade 4 IDHm astrocytoma for this study (Fig. 1).

Nine patients obtained their grade 4 diagnosis from *CDKN2A/B* deletion, 16 patients had tumors with morphological characteristics of WHO grade 4, and four patients had tumors with combined criteria. As seen in Fig. 1, there were 6 patients with morphological diagnosis without *CDKN2A/B* assessment. These patients were excluded from the subgroup comparisons but included in the description of the whole cohort.

### Public cohort

From an analysis of 249 patients from TCGA, 55 patients were included. In 38 cases, *CDKN2A/B* status was determined using methylation array data, in the remaining 17 cases genomic data served as basis for *CDKN2A/B* assessment. A *CDKN2A/B* deletion, without morphological WHO grade 4 alterations, was found in 26 cases, while 20 were classified as WHO grade 4 based solely on morphological criteria. The remaining nine cases had combined *CDKN2A/B* deletion and morphological WHO grade 4 criteria (Supplementary material Table 1).

### Characteristics of IDHm astrocytoma WHO grade 4

The most common symptoms at presentation were headache (51%) and seizures (49%). More demographic information and baseline characteristics are presented in Table 1.

**Table 1:** Characteristics of IDHm Astrocytoma grade 4 in the local cohort

Baseline characteristics	*n* = 35
Male, n (%)	20 (57)
Age, Mean (SD)	43 (15)
Symptoms, n (%)^a^	
Seizures	17 (49)
Headache	18 (51)
Motor deficit	5 (14)
Language deficit	6 (17)
Visual deficits	4 (11)
Main tumor location, n (%)	
Frontal	21 (60)
Temporal	7 (20)
Other	7 (20)
Preoperative tumor volume, Median ml (Q1, Q3)	65 (33, 114)
Contrast enhancing tumors, n (%)	18 (51)
KPS score preoperatively, n (%)	
< 70	1 (3)
70	9 (26)
80–100	25 (71)
Treatment following Stupp regimen, n (%)	29 (83)
*MGMT*, n (%)	
Methylated	19 (54)
Unmethylated	5 (14)
Missing	11 (31)
G-CIMP, n (%)	
High	23 (66)
Low	6 (17)
Missing	6 (17)

^a^Each patient could have several symptoms, therefore adding to more than 100%. KPS denotes Karnofsky Performance Status

All patients underwent brain tumor resection as part of their initial treatment, including one patient who required acute surgery. The median time from radiological diagnosis to surgery was 19 days, with no significant difference observed between subgroups (*p* = 0.57, Supplementary material Table 2). For first-line oncological management; 29/35 patients received treatment according to the Stupp regimen with concomitant temozolomide and radiation followed by adjuvant temozolomide. There was no significant difference in treatment strategy between the subgroups (*p* = 0.26 Supplementary material Table 2).

When comparing the different subgroups of astrocytomas WHO grade 4 with respect to baseline characteristics, as well as G-CIMP and *MGMT* status, there were no differences.

### Survival

**Fig. 2:**
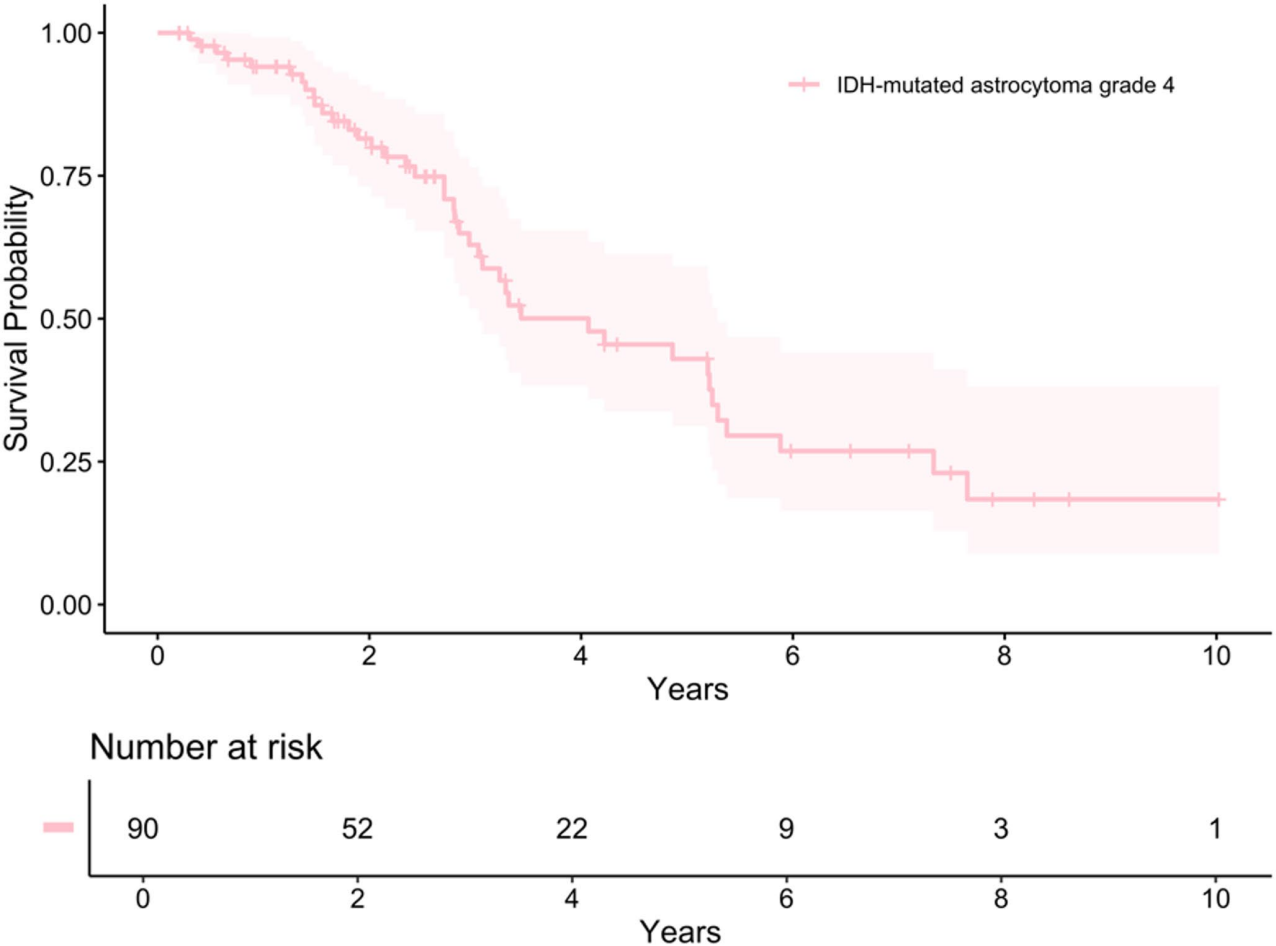
Survival of patients with an IDHm astrocytoma grade 4

The median OS for all the 90 IDHm astrocytomas WHO grade 4 patients of the combined cohort was 4.1 years (95% CI 3.0-5.3) (Fig. 2). There was no significant difference in survival between the local and the public cohort (median OS 5.2 vs. 3.2 years, *p* = 0.06). Patients who were operated at Sahlgrenska University Hospital and received treatment following the Stupp regimen (*n* = 29, 83%) had a median OS of 5.3 years (95% CI 3.3-not reached).

**Fig. 3:**
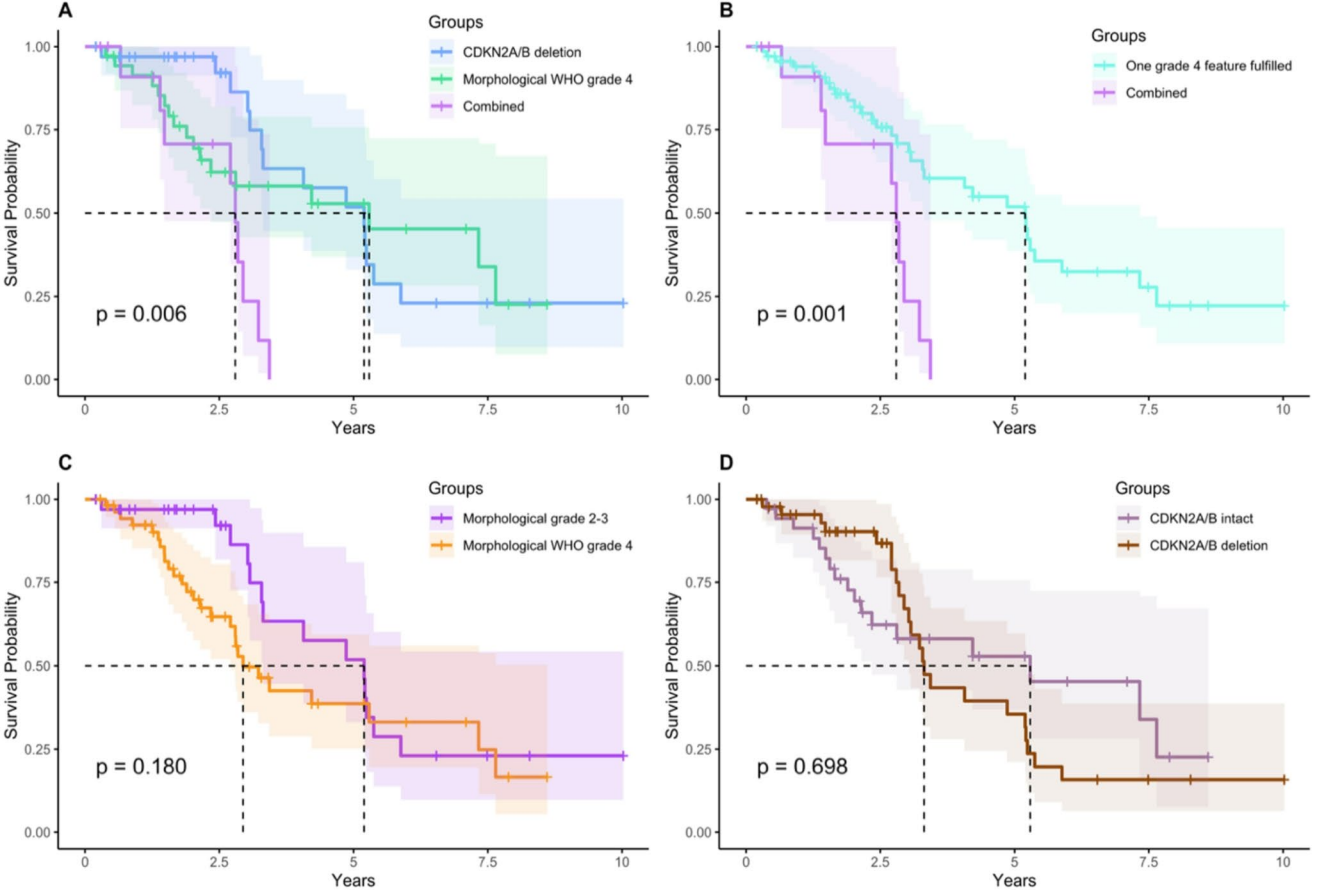
Survival analyses were conducted among different subgroups of IDHm astrocytoma grade 4. **A**) Survival comparison between molecular criteria (blue), morphological criteria (green), and combined criteria (purple). The subgroup with combined morphological and molecular WHO grade 4 criteria showed significantly worse survival than the other subgroups (*p* = 0.006). **B**) Comparison between tumors having only *CDKN2A/B* deletion or morphological WHO grade 4 features versus combined criteria **C**) Morphological WHO grade 4 astrocytomas compared to morphologically lower-grade astrocytomas harboring a *CDKN2A/B* deletion revealed no significant difference in survival (*p* = 0.18). **D**) Survival analysis of astrocytomas WHO grade 4 revealed no significant difference in survival between *CDKN2A/B* deleted tumors compared to those with intact *CDKN2A/B* status (*p* = 0.70)

The median survival in patients with *CDKN2A/B* deletion was 5.2 years (95% CI 3.3- not reached) compared to 5.3 years (95% CI 2.3- not reached) for the morphological subgroup, but only 2.8 years (95% CI 1.5- not reached) for the subgroup fulfilling combined criteria (*p* = 0.006) (Fig. 3A). In a sensitivity analysis, analyzing cohorts where *CDKN2A/B* status was assessed by DNA-methylation profiling, the survival difference remained (*p* = 0.002, Supplementary materials Fig. 2).

There were no significant differences in survival within astrocytomas WHO grade 4 when grouping patients only by morphology or *CDKN2A/B* deletion (Fig. 3C-D), as well as when comparing G-CIMP high tumors to G-CIMP low (Supplementary materials Fig. 2).

## Discussion

In this study, the median survival following a diagnosis of IDHm WHO grade 4 was 4.1 years. Survival was significantly worse when the tumor presented with both WHO grade 4 morphology and *CDKN2A/B* deletion. The clinical characteristics and treatment were similar between the different subgroups of reaching a diagnosis of IDHm astrocytoma WHO grade 4.

In patients with astrocytoma WHO grade 4 where morphological criteria were met, presence of *CDKN2A/B* deletion was associated with worse survival (combined subgroup). This is in line with some earlier studies [4, 11, 25], while contradicting others [2, 10]. The reason for these discrepancies remains a question for further research but a possible explanation could be the difference in methods used for assessment of *CDKN2A/B* status across studies. Methods used for *CDKN2A/B* assessment include methylation and SNP array technology, quantitative PCR and FISH [2, 4, 10, 11, 25]. To further complicate matters, the commonly used DNA methylation profiling to assess *CDKN2A/B* status lack consensus on appropriate cutoff for defining a homozygous deletion [11, 26]. Choice of method and cut-offs will contribute to different results. In this study we applied the commonly used, previously published, methylation-based method without exploring alternative cut-offs for *CDKN2A/B* deletion [11, 15]. However, multicenter studies using the same technology should be performed to confirm our results.

We observed no difference in survival between IDHm astrocytoma WHO grade 4 exhibiting *CDKN2A/B* deletion only compared to morphological criteria only. Existing literature has shown conflicting data on this topic. Evidence of worse survival has been reported in IDHm astrocytoma WHO grade 4 with diagnosis based upon *CDKN2A/B* deletion only compared to IDHm astrocytoma WHO grade 4 where diagnosis has been based upon morphology [8, 11]. But there are also reports of worse survival in astrocytoma grade 4 when the diagnosis was based on morphology, although this was not statistically significant [27]. Yet others have observed comparable survival in astrocytomas grade 4 regardless of diagnostic means [2]. The discrepancies may be explained by differences in treatment strategies among the study populations, where patients with only *CDKN2A/B* deletion have historically been treated as “lower-grade gliomas”, while those with morphological criteria typically have received a more aggressive treatment following the Stupp regimen. This is a limitation when retrospective reclassification is used, a phenomenon that affects most studies done to date. Some of the divergent findings can probably also be explained by the rarity of the diagnosis and the limited sample size in most series, making it prone to spurious findings. But the conflicting evidence across studies could also be influenced by lack of consideration for overlapping features. For instance, earlier studies have not systematically accounted for the double impact of a *CDKN2A/B* deletion in tumors with morphologically defined WHO grade 4 astrocytomas [8, 25, 27]. Such overlap could influence survival, emphasizing the need for future studies that integrate both molecular and morphological factors to provide a more comprehensive understanding of these tumors.

Identifying tumor characteristics that contribute to poorer survival is important, even in the absence of improved treatment options currently available for these patients. For instance, preclinical evidence suggests that CDK4/6 inhibitors, already approved for use in breast cancer, exhibit antitumor effects on IDHm astrocytomas harboring a *CDKN2A/B* deletion [28]. Our study suggests that patients who meet combined criteria with both morphological WHO grade 4 criteria and harbor a *CDKN2A/B* deletion are particularly good candidates for clinical trials evaluating promising experimental therapy, as their survival outcomes are notably worse. Also, if not considering the dual impact in trial design, the effects of experimental treatment may be exaggerated or reduced. Further, inhibitors of *IDH* mutations have shown promise in WHO grade 2 tumors [29], but no positive signals in monotherapy in tumors with contrast enhancement were seen in earlier studies [30, 31]. We speculate that this could be due to other oncogenic drivers, such as *CDKN2A/B* deletion coming into play. But subgroups with better expected survival, with solely morphological criteria, could potentially benefit from inhibitors of mutant *IDH* in mono- or combinational therapy.

### Strengths and weaknesses

The major weakness of this study is the limited population size which is a frequent issue in this tumor type since it is a rare form of cancer. To validate our observations, larger multicenter studies are encouraged.

Another limitation is the variability in image modalities used for tumor volume segmentation. This could exaggerate tumor volume in cases calculated from T2-weighted images since edema could have been segmented as tumor as well as lead to an underestimation of the volumes calculated from T1-weighted images since it is more difficult to account for non contrast enhancing (CE) volume on these modalities, especially when significant edema is present. However, only tumors in which most of their volume was CE were segmented in T1-modalities, so these tumors are not results of a smaller CE in a large non-enhancing lesion where we previously called such areas focus of malignant degeneration of low-grade glioma to a glioblastoma. For such tumors with a significant proportion of non-CE tissue, segmentation was performed using T2 to ensure accurate representation.

*CDKN2A/B* status in this study was determined using both methylation arrays and genomic data, when methylation data was missing. While this methodological variability may introduce some heterogeneity, it also enhances the clinical relevance of the findings, since diagnostic approaches differ across institutions, thereby increasing the generalizability of our results.

The strengths of this study include the population-based cohort and homogeneity of treatment across most patients, minimizing variability that could confound the results.

## Conclusion

IDHm astrocytoma WHO grade 4 diagnosed by simultaneous *CDKN2A/B* deletion and morphological criteria had a significantly worse survival than if diagnosis was based upon only one of the criteria. With this clinical difference in mind, the different subgroups should influence trial design as they have different prognosis and may even respond differently to therapies.

## Electronic supplementary material

Below is the link to the electronic supplementary material.

Supplementary Material 1

## Data Availability

Parts of the internal cohort can be made available upon reasonable request. The external data referenced in this paper is publicly available through TCGA (https://portal.gdc.cancer.gov).
