# Supplementary material for: CDKN2A/B status versus morphology in diagnosing WHO grade 4 IDH-mutated astrocytomas: what is the clinical relevance?
Source: J Neurooncol. 2025 May 20;174(2):449–58. doi: 10.1007/s11060-025-05078-x (PMC12208962; doi:10.1007/s11060-025-05078-x)

**Supplementary material**

**Table 1**: Describes the cohorts from which the patients in the present study were drawn, along with their morphological and molecular characteristics.

|  | | **Subgroups of IDHm astrocytoma grade 4 from the different cohorts** | | |
| --- | --- | --- | --- | --- |
|  | |  | Frequency | % |
|  | Sahlgrenska | *CDKN2A/B* deletion | 9 | 25.7 |
|  |  | Morphological WHO grade 4 | 16 | 45.7 |
|  |  | Morphological WHO grade 4 and uncertain *CDKN2A/B* status | 6 | 17.1 |
|  |  | Combined | 4 | 11.4 |
|  |  | **Total** | **35** | **100.0** |
|  | TCGA | *CDKN2A/B* deletion | 26 | 47.3 |
|  |  | Morphological WHO grade 4 | 20 | 36.4 |
|  |  | Combined | 9 | 16.4 |
|  |  | **Total** | **55** | **100** |
| T | **Total** | *CDKN2A/B* deletion | 35 | 38.9 |
|  |  | Morphological criteria | 36 | 40.0 |
|  |  | Morphological WHO grade 4 and uncertain *CDKN2A/B* status | 6 | 6.7 |
|  |  | Combined | 13 | 14.4 |
|  |  | **Total** | **90** | **100** |

**Table 2**: Characteristics of grade 4 astrocytomas when dividing into three different subgroups.

| **Characteristics between subgroups of astrocytoma grade 4 in local cohort** | | | | |
| --- | --- | --- | --- | --- |
|  | ***CDKN2A/B***  **deletion** | **Morphological WHO grade 4** | **Combined** | **P-value** |
|  | **N = 9** | **N = 16** | **N = 4** |  |
| Gender, Male (%) | 6 (66.7) | 6 (37.5) | 3 (75.0) | 0.247 |
| Age, Mean (SD) | 42 (10) | 42 (15) | 44 (21) | 0.964 |
| Preoperative tumor volume,  Median ml (Q1, Q3) | 108 (72, 151) | 61 (32, 117) | 69 (38, 97) | 0.393 |
| Symptoms, n (%) |  |  |  |  |
| Seizures | 6 (66.7) | 9 (56.3) | 2 (50.0) | 0.883 |
| Headache | 4 (44.4) | 8 (50.0) | 1 (25.0) | 0.873 |
| Cognitive impairment | 1 (11.1) | 4 (25.0) | 2 (50.0) | 0.334 |
| Minimum 1 focal deficit^a^ | 2 (22.2) | 6 (37.5) | 1 (25.0) | 0.853 |
| Main tumor location, n (%) |  |  |  | 0.587 |
| Frontal | 4 (44.4) | 11 (68.8) | 4 (100) |  |
| Temporal | 2 (22.2) | 2 (12.5) | 0 (0) |  |
| Parietal | 2 (22.2) | 3 (18.8) | 0 (0) |  |
| Insular | 1 (11.1) | 0 (0) | 0 (0) |  |
| Time from radiological diagnosis to surgery, median days (Q1, Q3) | 18 (15, 50) | 21 (10, 42) | 17 (10, 22) | 0.568 |
| G-CIMP high, n (%) | 8 (88.9) | 13 (81) | 2 (50) | 0.288 |
| *MGMT* Methylated, n (%) | 6 (66.7) | 9 (56.3) | 2 (50) | 0.272 |
| Total surgeries, n (%) |  |  |  | **0.047** |
| 1 | 5 (55.6) | 15 (93.8) | 3 (75.0) |  |
| 2 | 3 (33.3) | 0 (0) | 0 (0) |  |
| 3 | 1 (11.1) | 1 (6.3) | 1 (25.0) |  |
| Concomitant radiochemotherapy, n (%) | 6 (66.6) | 14 (87.5) | 4 (100) | 0.257 |
| Karnofsky preop, n (%) |  |  |  | 0.551 |
| <70 | 0 (0) | 1 (6.3) | 0 (0) |  |
| 70 | 1 (11.1) | 3 (18.8) | 1 (25.0) |  |
| >70 | 8 (88.9) | 12 (75.0) | 3 (75.0) |  |
| Karnofsky postop, n (%) |  |  |  | 0.109 |
| <70 | 0 (0) | 1 (6.3) | 0 (0) |  |
| 70 | 4 (44.4) | 4 (25.0) | 0 (0) |  |
| >70 | 5 (55.6) | 11 (68.8) | 4 (100) |  |

^a^Focal deficits included motor, language or visual deficit.

**Figure 1**: T1Gd (left) and T2-weighted/FLAIR (right) MRI images of all patients that were included in the subgroup analysis of preoperative tumor volume. The green boxes indicates which image modality that was used for each case. A1-A4) Tumors exhibiting both a CDKN2A/B deletion and morphological WHO grade 4 criteria. B1-B16) Morphologically defined WHO grade 4 astrocytomas without a CDKN2A/B deletion C1-C9) Astrocytomas classified as WHO grade 4 based solely on the presence of a CDKN2A/B deletion.


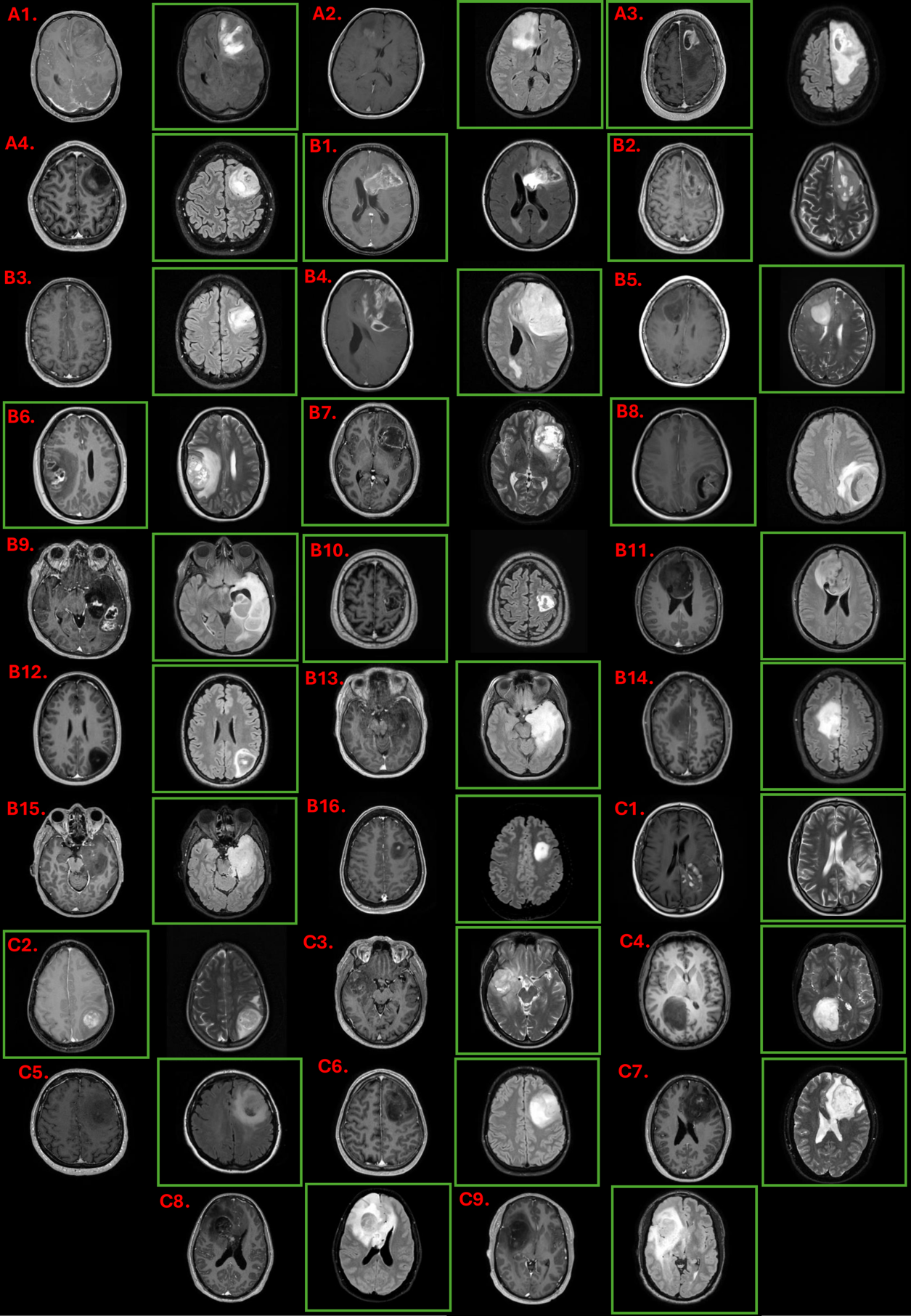


**Figure 2**: A) Survival of astrocytomas WHO grade 4 when only analyzing patients that have been operated at Sahlgrenska University Hospital. B) Survival of astrocytomas WHO grade 4 when *CDKN2A/B* assessment have been made using DNA methylation profiling. C) Survival when grouping patients based on their G-CIMP status.


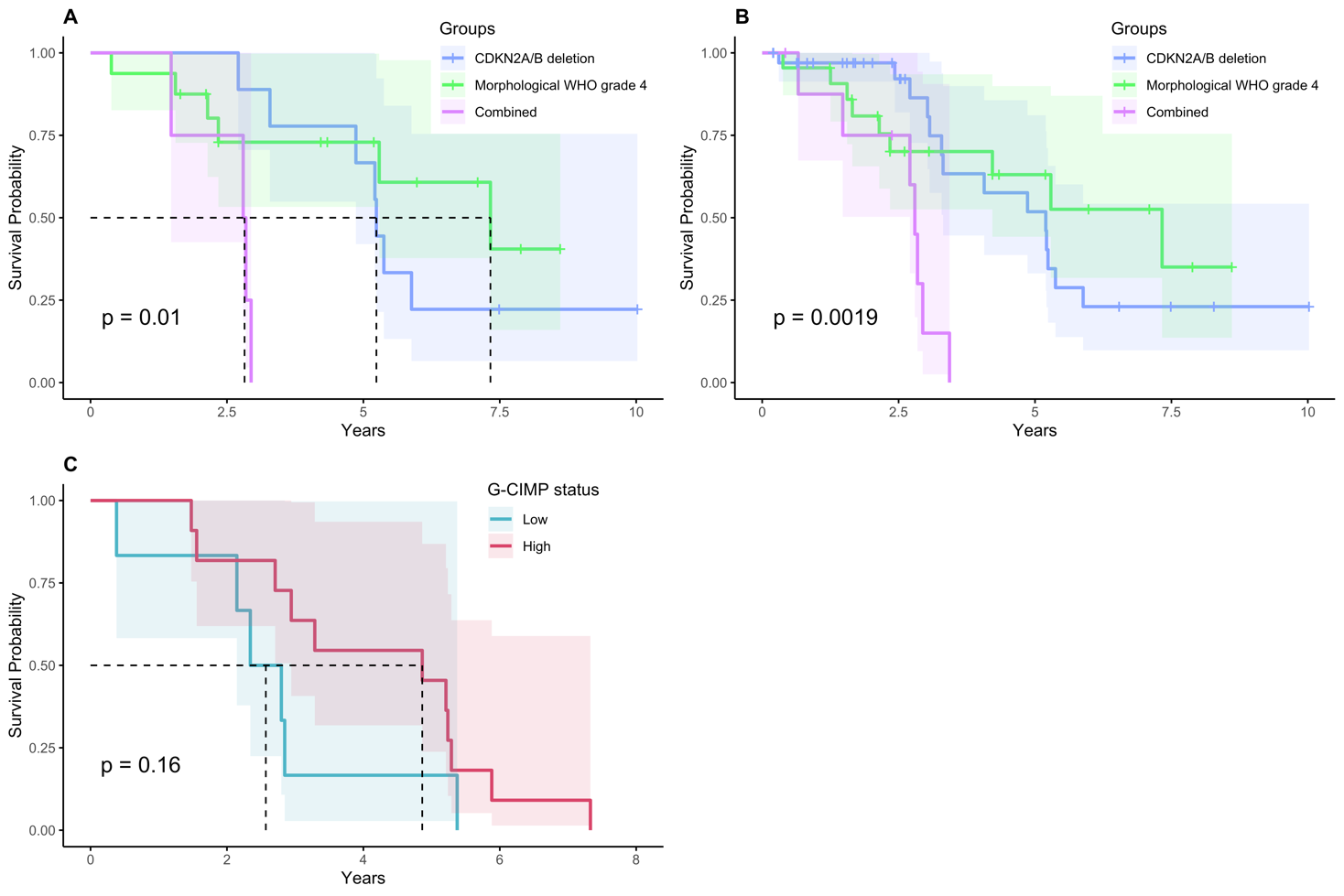

Supplement: Supplementary file 1 — Supplementary Material 1 [file 11060_2025_5078_MOESM1_ESM.docx]
